# Plain language communication as a priority competency for medical professionals in a globalized world

**Published:** 2018-05-31

**Authors:** Fiona Warde, Janet Papadakos, Tina Papadakos, Danielle Rodin, Mohammad Salhia, Meredith Giuliani

**Affiliations:** 1 Department of Radiation Oncology, Princess Margaret Cancer Centre, Ontario, Canada; 2 Cancer Education Research, Princess Margaret Cancer Centre, Ontario, Canada; 3 eLearning and Curriculum, Princess Margaret Cancer Centre, Ontario, Canada; 4 Department of Radiation Oncology, Brigham and Women’s Hospital/Dana Farber Cancer Institute, Massachusetts, United States; 5 Continuing Education and International Programs, The Michener Institute of Education at The University Health Network, Ontario, Canada

## Abstract

This brief report aims to highlight the impact of globalization – the international movement of goods, people, and ideas – on patient-provider communication in medical training and practice, and how the implementation of plain language communication training as a core competency for care providers can mitigate this impact. Globalization influences both patient and provider population diversity, which presents challenges with regard to patient-provider communication, particularly in cases of limited health literacy. Plain language communication - the delivery of information in a simple, succinct, and accurate manner - can help address these challenges. Training in plain language communication, however, is not a part of standard education for health care providers. Based on a synthesis of relevant literature pertaining to globalization, plain language communication, and medical education curricula, it is hoped that the information presented establishes the need for plain language communication as a core competency in medical education to enable providers to better meet the needs of an increasingly globalized health system.

## Globalization and medicine

Globalization is a phenomenon defined as the increasing international movement of people, ideas, and capital facilitated by political, economic, and technological advances.^1,2^ The changes globalization has brought to the world are widely felt, including in health care. From undergraduate medical education through to policy and regulation, these movements are influencing how medicine is taught and practiced.^3^ These changes are seen in the international import and export of medical school curricula, the increasing mobility of health professions students and practitioners, and a change in medical school structures – most notably in a shift towards a more profit-driven organizational model.^1,3,4^

Globalization has also impacted the diversity of populations physicians serve and the health system requirements needed to care for these people. Contributing to this are globalization’s facilitation of increased patient mobility^5^ and an increase in the global burden of non-communicable diseases (NCDs).^6,7^ The increased NCD burden is attributed in part to urbanization and spread of modifiable risk factors - such as tobacco use - resultant from globalization.^6,7^ Many of these NCDs are chronic conditions that result in patients spending greater time within the health care system and requiring them to participate more actively in decision making, symptom reporting, and self-management.^8^ This increased demand on patient involvement necessitates greater efforts in patient-provider communication to empower patients to successfully interact with the health care system.

## Patient-provider communication, health literacy, and globalization

Patient-provider communication is important for its role in facilitating patients’ active participation in their care, as well as influencing patient well-being and mitigating the effects of limited health literacy.^9^ Health literacy is defined as “the degree to which individuals have the capacity to obtain, process, and understand basic health information and services needed to make appropriate health decisions.”^10^ The prevalence of limited health literacy across the globe has been referred to as a “silent epidemic”^11^ because of its linkage to multiple forms of poor health outcomes, in addition to poor self-management and medication non-compliance.^12-14^ Limited health literacy has been a long-standing issue at the policy level which has consistently not been addressed,^15^ at great expense to the health care system. Eichler and colleagues reported in their systematic review that limited health literacy is responsible for 3-5% of health care system budget spending^16^ (about eight billion dollars in 2009). To address the barriers posed by health literacy, patient-provider communication must become a priority of health care organizations and systems given their combined impact on population health.^17,18^

Best practices for patient-provider communication are found in the literature,^10,13,17,19,20^ but limited work has been done to assess globalization’s influence on this area, and the resultant implications for care providers and their training.^3^ Canada’s diverse demographics, including large numbers of Indigenous, immigrant, and refugee populations, provides an excellent opportunity to examine this issue.^21^ Based on 2011 data from Statistics Canada, approximately 20% of the Canadian population was born outside the country, and this number is expected to increase to 25% by 2031.^21^ While the country has a highly educated population, The Canadian Council on Learning reports that 60% of Canadian adults have limited health literacy.^22^ This average is also highly regionalized, varying further by subpopulations within each region.^22^ Those subgroups with the lowest levels of health literacy are seniors, new Canadians, and unemployed persons; Canada’s Indigenous populations also rank low by these health literacy measures.^22^ Limited health literacy places patients’ health at risk, as well as reflects the high usage of health care resources by these groups.^9^ As such, limited health literacy is not just an individual problem, but a responsibility of the health care system and society as a whole.^15,23^

Our own experiences in Toronto, Canada, where patient populations are culturally and linguistically diverse, have clearly shown us the need to ensure medical trainees are skilled in plain language communication, which is the delivery of information in a simple, succinct, and accurate manner.^24,25^ Our aim with this report is to provide an overview of plain language communication and advocate for it to become a core competency for health care providers. We believe that requiring this competency at a policy level, integrating the relevant training into medical curricula, as well as providing continuing education programs for professionals already in practice, would further enable health care providers to meet the needs of our increasingly globalized world.

## Plain language communication

Plain language communication is utilized across multiple disciplines,^24^ and its value is supported by the research behind its development that draws from an equally diverse number of fields.^15^ This value is increasingly being recognized in health care applications for its role in mitigating the barriers posed by limited health literacy and enhancing patient safety.^15,24^ Plain language is defined as communication that can be understood the first time it is seen or heard,^24^ that uses succinct active-voiced grammatically correct complete sentences to better enable patients and caregivers to engage with information, using a more informal tone and common terms whenever possible.^15,25^ The table below illustrates how plain language review can enhance clarity (Table 1).

**Table 1:** Application of plain language in patient self-monitoring instructions

Before plain language review	After plain language review
You can self-monitor by observing any of the following signs that persist for 2 weeks or more: Unintentional weight loss Persistent cough Hematochezia Hematuria Profound fatigue	Report these signs to your oncologist (cancer doctor) if they last 2 weeks or more: Unplanned weight loss of more than 10 pounds Cough that won’t go away Blood in urine (pee) or stool (poo) New fatigue (feeling very tired)

The aim of this approach is not to oversimplify the health information itself but to structure and present it so that the message is simpler and therefore more accessible to the intended users.^15^ When done effectively, no essential information is lost while at the same time the material is presented in a manner that aims to enhance users’ comprehension and engagement.^15^ Those with intermediate or high health literacy equally benefit from plain language communication because in times of illness, pain, and distress it can be difficult to take in information.^26-28^ The AHRQ recommends a universal precautions approach to patient education on this basis, wherein all patients are assumed to have limited health literacy.^14^

There is a set of design principles for plain language materials; six of them are most important: logical organization, use of an introduction to outline content, writing using short sentences and paragraphs, layout that effectively utilizes white space, use of tables, and choice of typography.^25^ The Agency for Healthcare Research and Quality (AHRQ), and other highly reputable institutions, provide well-structured guidelines for development of resources using plain language communication.^25,29,30^ Each design principle listed above is rooted in evidence from the field of literacy. For example, since people with low literacy often read (decode) one word at a time they often forget preceding words by the time the reach the end of the sentence.^31^ The plain language practice of using short sentences and short paragraphs supports understanding by minimizing the effort required to understand key messages – and this also benefits those who read well.^31^ Plain language communication is not only for use in resource development, however, but is equally important in verbal interactions with patients.^19^

## Plain language communication in the Canadian health care setting

In the globalized Canadian context, use of the plain language communication approach in patient-provider communication resource development can decrease the barrier presented by English (or French as in Quebec) being the primary language of health care delivery, while being the second language of many Canadians.^32^ Forty-two percent of Canadian immigrants have reported “persistently poor” English-language proficiency after two years of living in Canada.^33^ Limited English proficiency (LEP) may pose a greater health risk than limited health literacy, as research has shown that individuals with LEP alone are significantly more likely to report poor health (41%) compared to individuals with limited health literacy alone (22%).^34^ As such, plain language communication’s use of common terms that are more likely to be understood by those with LEP and/or limited health literacy can help attenuate these increased health risks. In addition, given the increasing number of languages being encountered in Canadian medical settings,^35^ translated patient education materials using the same plain language communication principles can help to ensure that LEP patients have greater opportunity to understand health information. Evidence also shows that some LEP patients may have higher health literacy in their primary language.^32^ Based on the authors’ experience, back-translation of materials to English should be done to ensure that plain language was retained in the translated version as inadvertent revision away from plain language can occur during the process of translation by professional medical translators.

Language is also reflective of culture, as it is a main mode of cultural transmission.^32^ Defined by the Institute of Medicine, “Culture is the shared ideas, meanings, and values that are acquired by individuals as members of a society.”^32^ The importance of culture lies in the influence it has on how individuals relate to the health information they are presented with, and in the fundamental relationship they have with the concepts of health and illness.^32^ In the context of health literacy, culture includes the broad lens of “how people identify themselves and with whom they identify in terms of values, perceptions, and actions.”^36^ Culture can consequently be understood to impact individuals’ health literacy level,^29^ and so plain language communication, in its role in clear, precise messaging, must then be applied in a culturally sensitive manner. For example, Table 2 is a before and after example of a sentence that a patient interpreted through their lens as an active person interested in fitness and healthy eating and the culture around certain words they have developed as a result.

**Table 2:** Application of plain language in patient pre-operative instructions

Before plain language review	After plain language review
Prior to surgery patients are required to cleanse with this fluid.	The day of your surgery, wash your body with this liquid soap.

A healthcare provider shared the above example during a health literacy workshop with the authors. Due to the passive sentence structure and imprecision of the words “cleanse” and “fluid,” her patient misunderstood the instruction and actually drank the ‘fluid’ in preparation for their surgery instead of washing with it. The provider explained that since the patient was accustomed to the word “cleanse” being used in the context of healthy booster drinks they automatically interpreted cleanse as drink.

The use of idiomatic terms, wherein the meaning of the phrase cannot be determined based on the words within it, are culture-specific and can be difficult to interpret for those whose primary language the idiom is not derived from.^37^ While idioms may be seen as a way of making content relatable, their lack of plain language makes information less clear, less accessible, and open to misinterpretation.^25,31^ An example of a sentence using idioms before and after plain language review is shown in Table 3.

**Table 3:** Application of plain language in clarifying idiomatic instructions to patients

Before plain language review	After plain language review
Once you are discharged the ball is in your court for monitoring your blood glucose levels.	Once you leave the hospital, you will need to check your blood glucose levels. Check your blood glucose levels at least two times per day.

Being a time of unparalleled international movement of patients and providers - as well as pervasive mass media - there is currently an untold diversity of cultural experience based on the variety of patients’ life experiences.^1,32^ Thus preparing providers to be adaptable in their use and development of plain language communication skills and materials will be paramount.^15^

## Plain language communication as a competency of medical education

One of the greatest impacts of globalization is its facilitation of exchange of resources at speeds previously unimaginable, and its influence in promoting a profit-driven mentality.^3^ This is immediately evident in the health education sphere,^1^ wherein medical education institutions have increasingly embraced the globalized world’s economics-centred model in their organizational frameworks.^3^ Equally, the priorities of these institutions have also shifted towards the for-profit model, resulting in a greater commodification of both human and knowledge capital in the form of students, staff, and medical curricula.^3^ While medical education institutions have adapted to the economic changes brought on by the globalized world, the content of medical education has failed to keep pace with the changes brought on by globalization’s broader influences on health.^1,3,4,38^ With medical educators only recently beginning to address the concept of globalization, evidenced by the shift in language surrounding medical education towards globally-minded terminology,^3^ it is time for medical education institutions to re-evaluate their approach to training care providers to meet the needs of the increasingly globalized population they serve.^1^

Medical education has been reformed twice before in the previous hundred years, and is now requiring a third iteration to meet contemporary society’s health needs.^1,39^ In this reform, recognition of the medical education system’s role in training providers who can meet the needs of health system in which they practice, while maintaining a global perspective, is necessary.^1^ To achieve this, a systems based approach is needed as globalization has resulted in the increased interconnectedness of the health, education, and health education systems at multiple levels.^1^ At the institutional level, a competencies based approach in key areas, which are determined based on population need, is being proposed to reform medical education.^1^ Regardless of location, we posit that plain language communication be included as a core competency for medical education globally. In the Canadian context, The Royal College of Physicians and Surgeons of Canada sets out the core competencies expected of Canadian medical graduates and lays the foundation of the College’s standards for medical education at the system level.^40^ These are laid out in their CanMEDS Framework, which details the six key roles physicians must possess to achieve the unifying role of medical expert: Professional, Communicator, Scholar, Collaborator, Leader, and Health Advocate.^40^ The framework was most recently updated in October 2015, but did not include plain language communication as a competency under any of its roles.^40^

While applicable across each aspect of the framework, plain language communication could most easily be integrated into the Communicator role, which is defined in the CanMEDS 2015 Physician Competency Framework document: “As Communicators, physicians form relationships with patients and their families that facilitate the gathering and sharing of essential information for effective health care.”^40^ Adding plain language communication into the enabling competencies that underlie the third key competency listed for Communicators, “Share health care information and plans with patients and their families,”^40^ would modernize and vastly improve this CanMEDS role. Once included in these nationally recognized standards, Canadian medical schools would be obligated to update their curricula to include formation, education, and training in plain language communication.

More broadly, plain language communication could also be introduced into standard practice by engaging other licensing bodies, both nationally and internationally, to include it in their competency-based curricula. At the international level, The Saudi Commission for Health Specialties would be a prime example of where this could easily be implemented, as they have adopted the CanMEDS Framework^40^ for their own curriculum development.^41^ Beyond undergraduate medical education, plain language communication training must also be integrated into postgraduate and fellowship curricula. The Royal Australian and New Zealand College of Radiologists, whose latest curriculum update also integrated the CanMEDS Framework,^40^ is another example where in their use of a competencies-based approach, plain language communication training could be integrated under the Communicator role.^42^ The European Society for Therapeutic Radiation Oncology is another organization that utilizes a competencies-based approach, and so too could incorporate plain language communication into their clinical training.^43^ In implementing plain language communication more broadly, newly qualified providers will receive this training at multiple levels of their education, and be better equipped to practice in our increasingly globalized world.

In the current Canadian context this does raise the question, however, of how existing and foreign-trained practitioners would be engaged to add these competencies to their practice, as well as how the potential for conflict between new graduates and existing staff in how they are applied can be mitigated. Resistance has been noted within the medical community to the implementation of plain language communication. This can be attributed to lack of clarity as to what it is, and the benefits it provides not only to the patient, but the provider and health care system as well.^15^ Continuing education opportunities for existing practitioners may be useful in bridging this knowledge gap. Equally, for foreign-trained physicians who come to practice in Canada, additional consideration may be warranted as to how to engage and empower them with the requisite knowledge and skills to apply plain language communication. It is not enough to address the implementation of plain language communication only in the training portion of the Canadian health care system. The systems approach^1^ must be used to ensure broader implementation in order for this competency to become the gold standard in practice.

### Conclusion

In its effect of facilitating increased international exchanges of financial, human, and knowledge capital, globalization has largely been problematized for its impacts on health and medical education. However, it equally presents opportunity. The burgeoning interdependence resultant from this phenomenon can be leveraged to increase and encourage opportunities for developing collaborative solutions to the issue of establishing new standards of professional competencies, such as plain language communication, that will enhance providers’ abilities to meet the needs of quickly evolving health systems and the populations they serve.^1^ At the core of these competencies needs to be a focus on the social and ethical responsibilities^38^ medical education has to the population it serves. - as for better or worse, medical education institutions, the students they train and the populations they serve, are effected by globalization.^3^
